# Cytarabine induces cachexia with lipid malabsorption via zippering the junctions of lacteal in murine small intestine

**DOI:** 10.1016/j.jlr.2023.100387

**Published:** 2023-05-16

**Authors:** Mi-Rae Park, Hye-Jin Lee, Hye-Min Jang, Nam Hoon Kim, Jun-Seok Lee, Yong Taek Jeong, Inho Kim, Sang-Hyun Choi, Kwan Sik Seo, Dong-Hoon Kim

**Affiliations:** 1 Department of Pharmacology, Korea University College of Medicine, Seoul, Republic of Korea; 2 Department of Biomedical Sciences, Korea University College of Medicine, Seoul, Republic of Korea; 3 Division of Endocrinology and Metabolism, Department of Internal Medicine, Korea University College of Medicine, Seoul, Republic of Korea; 4 Department of Internal Medicine, Seoul National University College of Medicine, Seoul National University Hospital, Seoul, Republic of Korea; 5 Department of Rehabilitation Medicine, Seoul National University Hospital, Seoul, Republic of Korea

**Keywords:** cytarabine, triglycerides, metabolic dysfunction, cachexia, malabsorption, small intestine, lymphatic endothelial cell, lacteal

## Abstract

Chemotherapy-induced cachexia causes severe metabolic abnormalities independently of cancer and reduces the therapeutic efficacy of chemotherapy. The underlying mechanism of chemotherapy-induced cachexia remains unclear. Here we investigated the cytarabine (CYT)-induced alteration in energy balance and its underlying mechanisms in mice. We compared energy balance-associated parameters among the three groups of mice: CON, CYT, and PF (pair-fed mice with the CYT group) that were intravenously administered vehicle or CYT. Weight gain, fat mass, skeletal muscle mass, grip strength, and nocturnal energy expenditure were significantly lowered in the CYT group than in the CON and PF groups. The CYT group demonstrated less energy intake than the CON group and higher respiratory quotient than the PF group, indicating that CYT induced cachexia independently from the anorexia-induced weight loss. Serum triglyceride was significantly lower in the CYT group than in the CON group, whereas the intestinal mucosal triglyceride levels and the lipid content within the small intestine enterocyte were higher after lipid loading in the CYT group than in the CON and PF groups, suggesting that CYT inhibited lipid uptake in the intestine. This was not associated with obvious intestinal damage. The CYT group showed increased zipper-like junctions of lymphatic endothelial vessel in duodenal villi compared to that in the CON and CYT groups, suggesting their imperative role in the CYT-induced inhibition of lipid uptake. CYT worsens cachexia independently of anorexia by inhibiting the intestinal lipid uptake, via the increased zipper-like junctions of lymphatic endothelial vessel.

Body weight is regulated by a balance between energy intake and expenditure (1, 2), which is crucial for successful treatment of cancer patients. Cachexia, a wasting syndrome characterized by the involuntary loss of body weight and skeletal muscle mass with or without fat mass, is frequently observed in cancer patients (3, 4). Cachectic cancer patients show poorer survival rate and prognosis than those with maintained body weight (5, 6, 7, 8). The wasting of adipose tissue contributes to weight loss during cachexia and is associated with low survival rate in cancer patients (9, 10).

Multiple factors, including tumor, tumor-derived secretory molecules, and chemotherapy, are involved in the development and progression of cachexia in cancer patients (11, 12, 13). Emerging evidences show that chemotherapy with cisplatin, 5-fluorouracil, irinotecan, or leucovorin induces cachexia independently of tumor by disturbing energy balance in vivo (14, 15). Moreover, some chemotherapeutic agents, including daunorubicin, etoposide, and cisplatin, cause cachexia in healthy animal models (16, 17, 18). Thus, chemotherapy greatly contributes to cachexia development during cancer treatment (16, 19).

Cytarabine (CYT), a pyrimidine antimetabolite that inhibits DNA synthesis in the S-phase of the cell cycle, has been widely used as a standard chemotherapy for leukemia (20, 21). Individual or coadministration of CYT with chemotherapeutic agents via various routes significantly decreases body weight in xenograft mouse model or healthy mice (22, 23, 24). Moreover, in cancer patients, the combined treatment with CYT and interferon causes weight loss and is discontinued more frequently than the treatment with interferons alone (25). Therefore, CYT administration may hinder the maintenance of energy balance during the treatment, which is important to conserve the therapeutic efficacy and the quality of life of cancer patients (2, 26). The adverse effects of chemotherapy have been hypothesized to affect the energy balance in humans and animals (21, 27). However, the precise effect of chemotherapy on energy balance and its underlying mechanisms remain elusive.

In this study, we established a mouse model of CYT-induced cachexia and investigated the energy balance and underlying mechanisms of the CYT-induced cachexia using pair-fed (PF) mice as controls. We found that the zipper-like junctions of lymphatic endothelial cells (LEC) of the small intestinal villi might contribute to the exacerbation of CYT-induced cachexia via inhibition of lipid uptake independently of anorexia in mice. Therefore, our findings highlight the critical role of lacteal in CYT-induced cachexia.

## Materials and methods

### Experimental model

Ten-week-old C57BL/6 male mice (Orient Bio, Seongnam, Republic of Korea) and *Lgr5-EGFP-IRES-creERT2* mice (28) obtained from Jackson Labs were housed individually with free access to chow diet and water ad libitum at 22°C, 60% humidity, and a 12:12 light:dark cycle. After 1 week of adaptation, the mice were intravenously administered CYT (100 mg·kg^−1^ body weight, JW Pharmaceutical Corp., Seoul, Republic of Korea) once a day for four consecutive days (9–11 AM), while the control (CON) and PF groups were intravenously administered saline (vehicle, 2 μl·g^−1^ body weight) or saline in combination with CYT, respectively. The dosage of CYT was determined based on previous studies (29, 30). The interventions resulted in the successful establishment of a murine model demonstrating dose-dependent anorexia and a decrease in weight gain and fat mass. Daily body weight and food consumption were measured, and the PF group was given the average amount of food consumed by the CYT group. All the mice were sacrificed a day after the last injection and fasted for 2 h prior to being sacrificed. All the experimental procedures were performed under the ethical approval guidelines issued by the Institute for Basic Science funded by the Ministry of Science and the Institutional Animal Care and Use Committee (authorization no. KOREA-2018-0036, KOREA-2023-0005) and ARRIVE (Animal in Research: Reporting In Vivo Experiments) (31). The animals were maintained in specific pathogen-free animal facilities authorized at Korea University College of Medicine (authorization no: N. 127/2012-A).

### Body mass profiling using NMR

TD NMR analyzer (Bruker Corporation, MA) was used to measure the lean and fat mass of mice on the last day of the treatment.

### Muscle grip strength test and indirect calorimetry

The maximum forelimb grip strength of the mice was measured by the grip strength test (Bioseb, FL). The mice were placed in metabolic cages connected to indirect calorimetry system combined with gas analyzer (Harvard Apparatus, MA) to measure O_2_ consumption and CO_2_ production to determine the energy expenditure (EE).

### Real-time quantitative polymerase chain reaction

TRIzol™ reagent (Invitrogen, Thermo Fisher Scientific Corp., MA) was added to the frozen tissue, which was homogenized using a tissue homogenizer (BioSpec Products Inc., OK). Isopropyl alcohol (Duksan Pure Chemicals Co., Ltd., Ansan, Republic of Korea) was added to the homogenized tissue, and it was subjected to centrifugation for 10 min of 12,000 *g* at 4°C. The supernatant was mixed with chloroform (Merck Millipore, MA), and total RNA was extracted as per the manufacturer’s protocol (Invitrogen, Thermo Fisher Scientific Corp., MA). The isolated RNA was dissolved in diethyl pyroprocabonate-treated RNase-free water (Welgene Inc., Gyeongsan, Republic of Korea). The complementary DNA was synthesized using 1 μg of the total RNA with the iScript cDNA synthesis kit (Bio-Rad Laboratories, Inc., CA). Gene expression was measured using SensiFAST SYBR Lo-ROX kit (Meridian Bioscience, TN) or TaqMan probes and TaqMan gene expression master mix (Applied Biosystems, Invitrogen, Thermo Fisher Scientific Corp., MA) on an Applied Biosystems 7500 real-time PCR Instrument System.

### Lipid analysis

Snap frozen mucosa was lysed with RIPA lysis buffer (Santa Cruz Biotechnology, Inc., CA), and the lipids were isolated from mucosa. The separated lipid-containing layer was dried with 2% Triton X-100 in chloroform under a laminar air flow hood. The dried mucosal lipid samples were dissolved in deionized water. Mice feces were collected every day during the experiment. Fecal lipids were extracted using the Folch method, and the extracted lipids were weighed after drying for 3 days. Briefly, 5 ml of saline was added to 1 g of powdered mice feces and mixed thoroughly. Equal volumes of the chloroform-methanol (2:1, v/v) solution were added to the saline-feces homogenate. The mixture was then vortexed and subjected to centrifugation for 10 min of 1,000 *g*. The resultant layer of lipids dissolved in the mixture was isolated, dried under hood, and weighed on a microscale. The concentration of triglyceride (TG), total cholesterol, and free fatty acids was determined using TG determination reagent (MilliporeSigma, MO), free glycerol determination reagent (MilliporeSigma), cholesterol quantitation kit (MilliporeSigma), and free fatty acid quantitation kit (MilliporeSigma) according to the manufacturer’s instructions. Protein concentration was estimated using the PierceTM BCA protein assay kit (Thermo Fisher Scientific Corp., MA) as per the manufacturer’s protocol, and the concentrations of TGs and proteins were measured using a micro plate reader (Molecular Devices, LLC., CA). Mucosal lipid levels were normalized to the protein concentration.

### Glycogen measurements

Snap frozen liver and skeletal muscle were homogenized in ice-cold H_2_O (10 μl·mg^−1^ tissue). The homogenates were boiled for 5 min, followed by centrifugation for 5 min of 13,000 *g* at 4°C, and the supernatants were collected. Glycogen levels were measured using a glycogen assay kit (MAK016, MilliporeSigma) according to the manufacturer’s protocol, and the concentration was normalized to the total protein amount, determined using the PierceTM BCA protein assay kit (Thermo Fisher Scientific Corp., MA).

### Measurement of serum AST and ALT levels

The concentration of serum aspartate aminotransferase (AST) (MAK055, MilliporeSigma) and alanine aminotransferase (ALT) (MAK052, MilliporeSigma) were determined using detection kits according to the manufacturer’s instructions.

### Oral fat tolerance test

The mice were administered olive oil (10 μl·g^−1^ body weight, MilliporeSigma) by oral gavage after a 2 h fast. Blood was collected at 0, 1, 2, 3, and 5 h post the lipid loading using capillary tubes from the tail vein with a small cut. Serum samples were prepared and stored at −80°C for lipid analysis. The area under the curve (AUC) was calculated by integrating the values during the entire experiment time, 0–5 h.

### Oil red O staining

Intestine was isolated 24 h after oral fat tolerance test (oFTT). The flushed intestine was dissected to obtain 6 cm of duodenum (at a distance of 1 cm from the stomach) and 9 cm of jejunum (at a distance of 15 cm from the stomach), fixed with alcoholic zinc formalin (Anatech USA, NV) overnight, and immersed in 30% sucrose/PBS solution. The cryopreserved tissue was embedded in Tissue-Tek OCT compound (Sakura Finetek USA, Inc., CA) for cryo-sectioning. OCT-embedded duodenal and jejunal tissues were sectioned (thickness: 5 μm) with a cryostat microtome (Leica Biosystems, Wetzlar, Germany). After 1 h of air-drying, the sections were fixed with ice-cold 10% formalin (MilliporeSigma) for 5 min and dried for 1 h. The slides were then placed in absolute propylene glycol (Junsei Chemical Co., Ltd., Tokyo, Japan) for 2 min and stained in prewarmed oil red O (ORO) solution for 8 min at 60ºC in an oven. After differentiation in 85% propylene glycol solution for 2 min, the slides were rinsed in distilled water twice and stained with hematoxylin for 10 s. Subsequently, the slides were washed thoroughly in running tap water and mounted with an aqueous medium.

### FITC-dextran permeability assay

To investigate the intestinal paracellular uptake through leaky junctions, FITC-dextran permeability assay was performed (32). The mice were orally administered approximately 4 kDa FITC-dextran (MilliporeSigma) at a concentration of 44 mg/100 g body weight after a 16 h fast, and the blood samples were collected after 4 h. The isolated serum was diluted five times with PBS, and fluorescence was detected at 528 nm using a micro plate reader (Molecular Devices, LLC.).

### Gastrointestinal motility measurements

Whole bowel transit time was assessed by using 150 μl of 6% (w/v) carmine red dye (Sigma-Aldrich) dissolved in 0.5% methyl cellulose solution. After oral administration, mice were placed in individual cage with white bottoms and checked at 15 min intervals through 8 h until red fecal pellets were observed. In separate studies, proximal intestinal motility was evaluated by oral administration of 100 μl of FITC-dextran (average molecular weight 70 kDa, MilliporeSigma) dissolved in PBS at a concentration of 2 mg/ml. Mice were euthanized with a ketamine/xylazine cocktail and sacrificed 2 h after administration. The gastrointestinal (GI) tract was removed without the removal of luminal contents and then frozen at −80°C. To analyze the distribution of FITC-dextran within the GI tract, the GI tract was divided into 12 sections as previously described (33). Thawed segments were flushed with 1 ml of ice-cold PBS and centrifuged at 10,000 *g* for 5 min at 4°C. Fluorescence of 10-fold diluted supernatant samples was quantified using an EnVision multimode plate reader (PerkinElmer).

### Immunostaining

For immunostaining of the whole-mount small intestinal tissue sample, transcardial perfusion was performed with 4% paraformaldehyde (PFA; Merck Millipore, MA), following anesthesia administration. The PBS-flushed small intestine was harvested as described in the preceding section and cut longitudinally, and the tissues were pinned on silicon plate to expose the lumen. After washing with PBS, the samples were postfixed with 4% PFA for 2 h at 4°C. After several washes with PBS, subsequent dehydration was performed with 10% sucrose in PBS for 2 h, followed by 20% sucrose and 10% glycerol in PBS overnight at 4°C. The dehydrated samples were blocked with 5% goat serum and 1% BSA in 0.5% Triton X-100 in PBS for 1 h and incubated with primary antibodies, which were diluted in the blocking solution, overnight at 4°C. After several washes with wash buffer (0.3% Triton X 100, MilliporeSigma, in PBS), the samples were incubated with secondary antibodies, which were diluted in the blocking buffer, in the dark for 2 h at 25°C. The samples were washed with wash buffer and mounted with aqueous mounting solution.

### Western blotting

The tissue was homogenized in RIPA lysis buffer system with a protease inhibitor cocktail and phosphate inhibitors (Santa Cruz Biotechnology, Inc., CA). The protein concentration was determined using the BCA protein assay kit (Thermo Fisher Scientific Corp., MA). The protein lysates were separated by SDS-PAGE and electroblotted onto PVDF membranes. The membranes were then blotted with primary antibodies; antiphospho-VEGFR2 (Cell Signaling Technology, MA; rabbit monoclonal, Cat # 2478; and polyclonal, Cat # 2471; both diluted 1:1,000), antiphospho-AKT (Cell Signaling Technology, MA; polyclonal, Cat #9271; diluted 1:1,000), anti-VEGFR2 (Cell Signaling Technology, MA; rabbit monoclonal, Cat #2479; diluted 1:1,000), anti-AKT (Cell Signaling Technology, MA; polyclonal, Cat #9272; diluted 1:1,000), and GAPDH (Cell Signaling Technology, MA; rabbit monoclonal, Cat #2118; diluted 1:1,000), followed by a horseradish peroxidase-conjugated secondary antibody (Cell Signaling Technology, MA; Cat #7074, diluted 1:1,000). Protein bands were visualized by chemiluminescence using a Bio-Rad ChemiDoc Imaging Systems with PicoEPD Western reagent kit (ELPIS BIOTECH, Inc., Daejeon, Korea).

### Histological analysis

The micrographs were obtained using Zeiss LSM 900 (Carl Zeiss AG, Jena, Germany). The primary and secondary antibodies used in the immunostaining are as follows: anti-LYVE-1 (AngioBio., CA; rabbit polyclonal; Cat # 11-034, diluted 1:400); anti-VE-cadherin (R&D Systems, MN; goat polyclonal; Cat # AF1002; diluted 1:200); Alexa Fluor 488- and Alexa Fluor 555-conjugated anti-rabbit (Invitrogen; Cat # A11008; diluted 1:1,000); and anti-goat (Invitrogen; Cat # A32816; diluted 1:1,000) antibodies. All the antibodies used in our study were validated for specific applications. Detailed description of magnification is indicated in the figure legends.

### Morphometric analysis

Morphometric measurement was conducted using the NIH-ImageJ software (available at https://imagej.nih.gov/ij/) after the processing of raw imaging data with the Zen software (Carl Zeiss). The VE-cadherin^+^ junctions were quantified within the LYVE1^+^ lacteal. The pattern of junctions in lacteals was analyzed as described previously (34). The zipper-like junctions were defined as continuous junctions at cell-to-cell borders with elongated shape, whereas the button-like junctions were identified as discontinuous junctions, which are not parallel to cell-to-cell borders.

### Electron microscopy

To observe ultrastructure of the small intestinal enterocytes, ultrathin sections of the duodenum were prepared with an ultramicrotome (Leica Biosystems). Briefly, the harvested samples were fixed with 2% PFA and 2.5% glutaraldehyde in 0.1 M phosphate buffer (pH 7.4) and fixed with 2% osmium tetroxide after several washes with PBS. After dehydration with increasing concentrations of ethanol and infiltration with propylene oxide, resin embedding was performed. The ultrathin sections were then double-stained with uranyl acetate, and lead citrate and images were obtained using a transmission electron microscope (Hitachi, Ltd., Tokyo, Japan) with 80 kV accelerating voltage.

### Statistical analysis

All statistical analyses were performed using GraphPad Prism 6.0 software (GraphPad Software, Inc., CA). Data are represented as mean ± SEM and were analyzed using two-way ANOVA followed by Bonferroni’s post hoc test or one-way ANOVA, followed by Tukey’s post hoc test where appropriate. *P* values less than 0.05 were considered statistically significant.

## Results

### CYT induces cachexia in mice

**Fig. 1:**
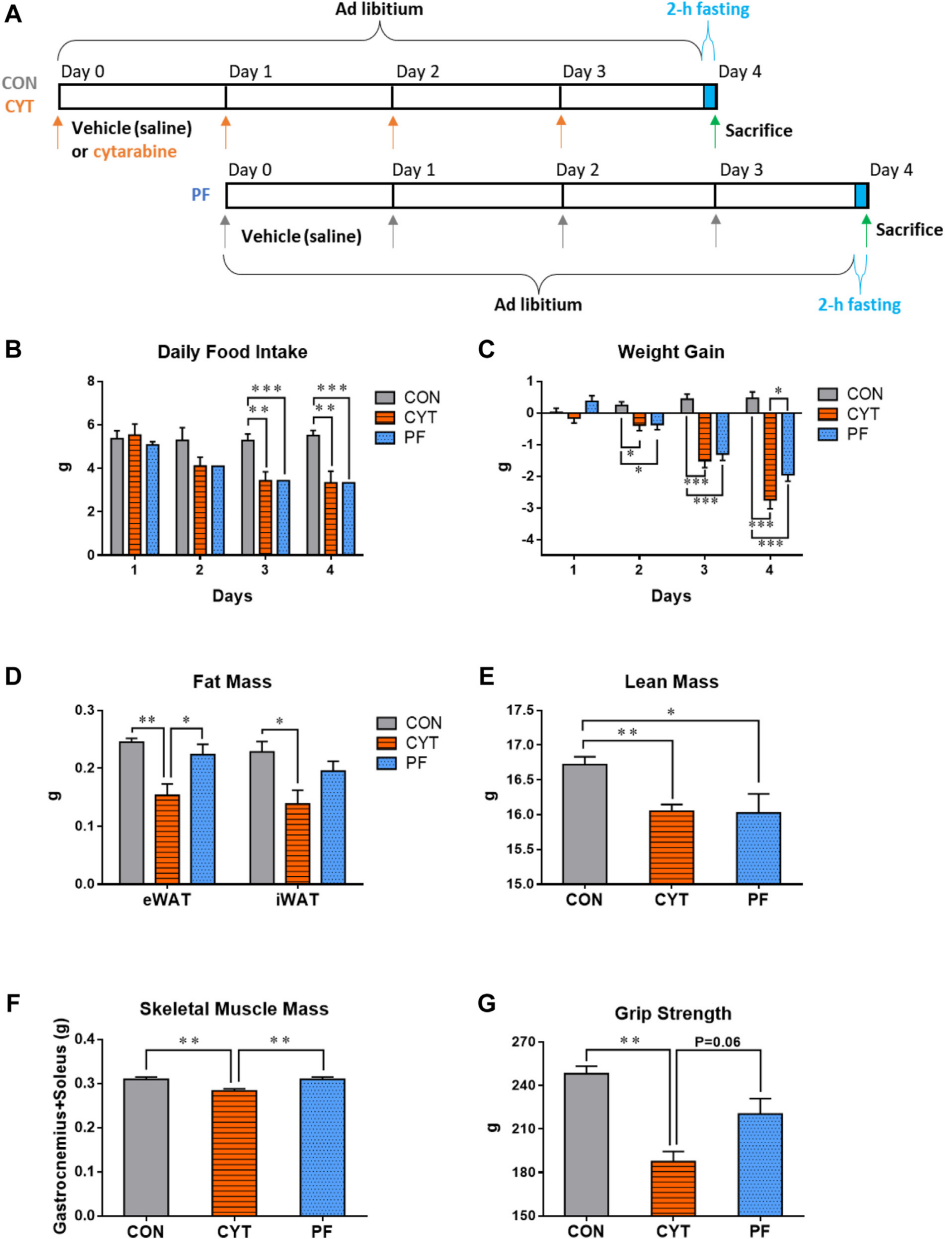
Cytarabine induces cachexia independently of the anorexia-induced weight loss. A: Schematic representation of the experimental timeline and dosing schedule. B: Daily food intake during the treatments. C: Changes in weight gain. D: Comparison of epididymal white adipose tissue (eWAT) mass and inguinal white adipose tissue (iWAT) mass among the indicated groups after treatment (day 4). E, F: Comparison of lean mass (E) and gastrocnemius and soleus muscle mass (F) among the indicated groups after treatment (day 4). G: Grip strength at day 4 of the treatments. ∗*P* < 0.05, ∗∗*P* < 0.01, and ∗∗∗*P* < 0.001. Data in (B–G) are represented as mean ± SEM. Data in (B, C) were analyzed using two-way ANOVA followed by Bonferroni’s post hoc test, and data in (D–G) were analyzed using one-way ANOVA between subjects followed by Tukey’s post hoc test. CON, control group treated with vehicle; CYT, group treated with cytarabine; PF, group pair-fed with the CYT group and treated with vehicle.

To investigate the effects of CYT on the regulation of energy balance, we established the PF group (PF with the CYT group and treated with vehicle) and compared the changes in the energy balance components among the three groups (Fig. 1A). The appetite of the CYT group was significantly less on days 3 and 4 than that of the CON group (−35% and −39%, respectively), and it was comparable between the CYT and PF groups throughout the experiment (Fig. 1B). Consistently, a significant weight loss was observed in the CYT and PF groups from day 2 to the end of experiment compared to that in the CON group. Moreover, weight gain was significantly lower in the CYT group (−11.08 ± 1.08%) than in the CON (1.86 ± 0.8%) and PF (−7.90 ± 0.77%) groups on day 4, indicating lower metabolic efficiency of CYT group than that of the PF group (Fig. 1C). Furthermore, the epididymal white adipose tissue (eWAT) mass and inguinal white adipose tissue mass of the CYT group was significantly lower than those of the CON group. Additionally, the eWAT mass of the CYT group was significantly lower than that of the PF group (Fig. 1D).

Lean mass was also significantly lower in the CYT and PF groups than in the CON group on day 4 (Fig. 1E). However, the mass of gastrocnemius and soleus muscle was considerably lower in the CYT group than in the CON and PF groups on day 4 (Fig. 1F), suggesting that CYT decreased the muscle mass independently of the anorexia-induced loss of muscle mass. Consistently, the grip strength test revealed that the muscle strength was lower in the CYT group than in the CON group (Fig. 1G). Therefore, the administration of CYT might induce cachexia (>5% weight loss) accompanied by loss of both fat and skeletal muscle masses via a mechanism different from that of the anorexia-induced weight loss in mice.

### CYT decreases EE in mice

**Fig. 2:**
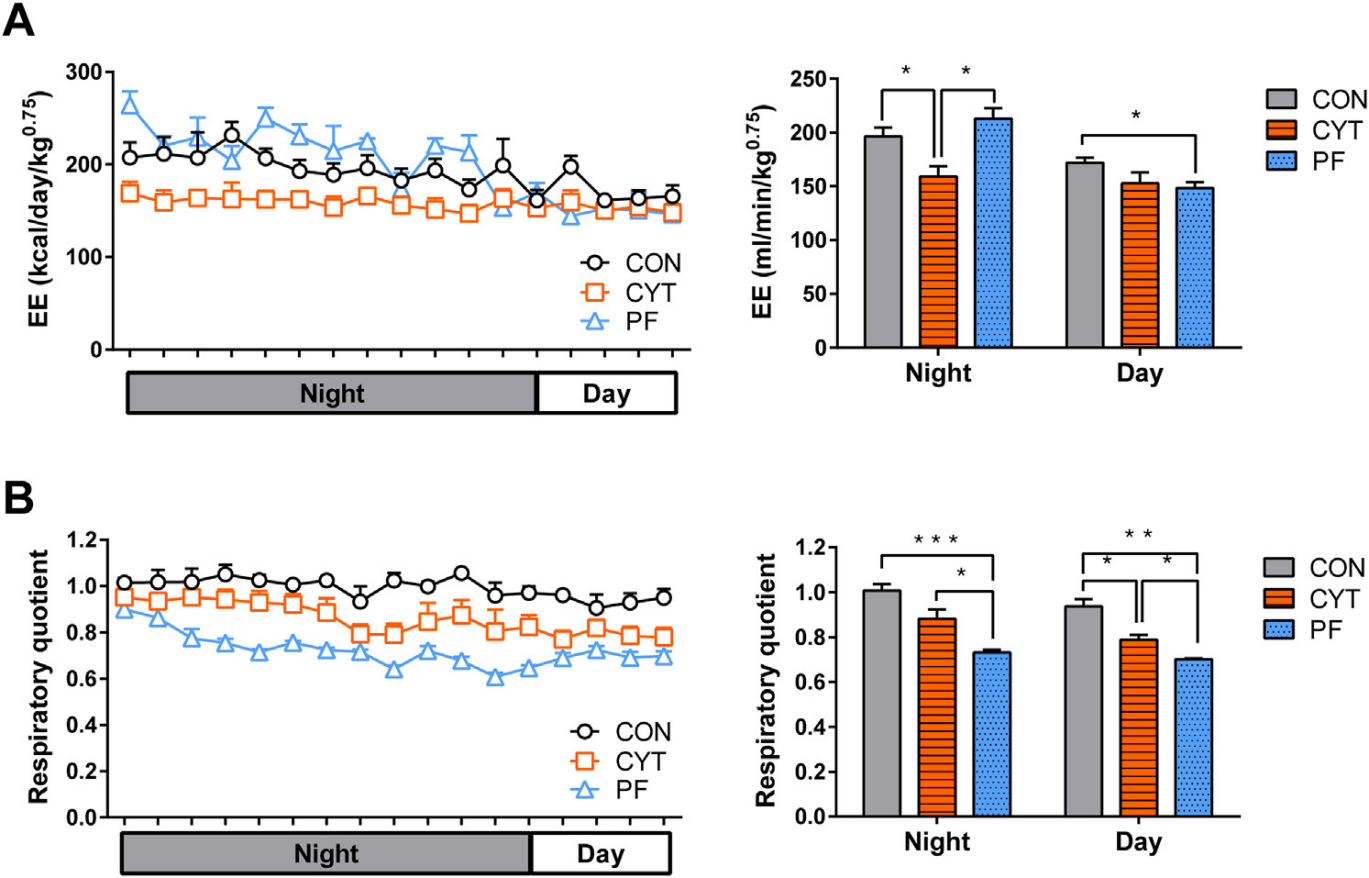
Cytarabine decreases energy expenditure in mice. A: Real-time monitoring curve of the energy expenditure (EE, left panel) and quantification of the expenditure (right panel) at day and night after vehicle or cytarabine treatment (day 4). B: Real-time monitoring curve of respiratory quotient value (left panel) and quantification of the same (right panel) at day and night after vehicle or cytarabine treatment (day 4). ∗*P* < 0.05, ∗∗*P* < 0.01, and ∗∗∗*P* < 0.001. Data in (A, B) are represented as mean ± SEM. Left panels of (A) and (B) were analyzed using two-way ANOVA followed by Bonferroni’s post hoc test, while right panels of (A) and (B) were analyzed using one-way ANOVA followed by Tukey’s post hoc test. *n* = 3–4 mice/group. CON, control group treated with vehicle; CYT, group treated with cytarabine; PF, group pair-fed with the CYT group and treated with vehicle.

To investigate whether changes in EE contributed to the CYT-induced weight loss in mice, we compared the oxygen consumption and respiratory quotient (RQ) on day 4 among the three groups. The nocturnal EE was significantly lower in the CYT group than in the CON and PF groups, despite the greater weight loss demonstrated by the CYT group than that by the CON and PF groups (Fig. 2A). Furthermore, RQ was significantly higher during the nocturnal period in the CYT group than in the PF group, whereas it was comparable between the CON and CYT groups (Fig. 2B). Two hours of fasting blood glucose levels and glycogen contents in liver and skeletal muscle were comparable among the three groups (Fig. S1A–C). These results indicated that the CYT group consumed less lipid as a fuel, which was different from the PF group.

Additionally, the brown adipose tissue (BAT) mass was not significantly different among the experimental groups (Fig. S1D). Consistent with the change in EE, BAT *Ucp1* expression was significantly lower in the CYT group than in the CON and PF groups. Additionally, eWAT *Ucp1* expression was significantly lower in the CYT group than in the CON and PF groups, suggesting the CYT-induced decrease in EE (Fig. S1E).

### CYT reduces the use of lipids as an energy source in mice

**Fig. 3:**
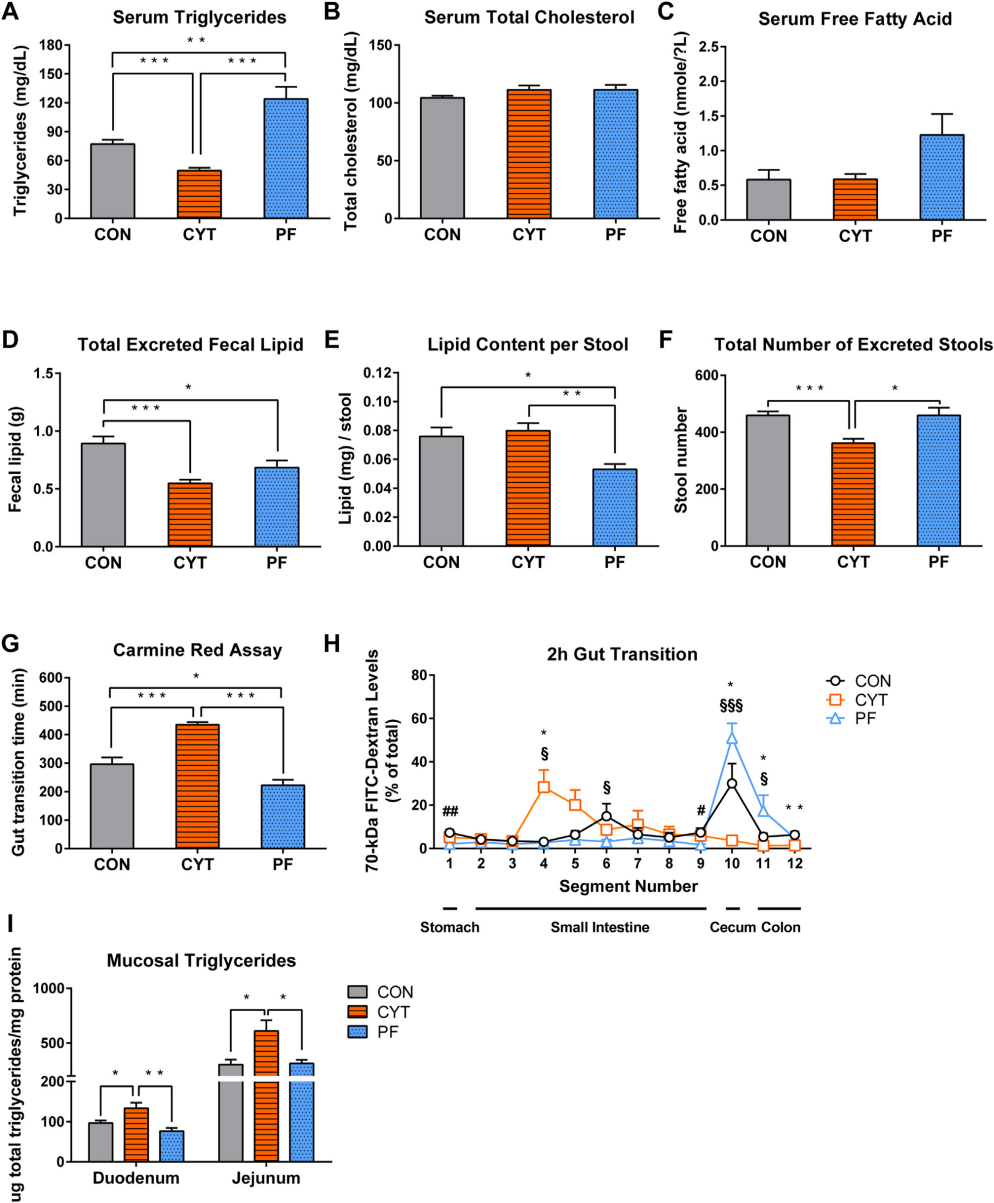
Cytarabine reduces serum triglyceride levels and increases lipid accumulation within the small intestinal mucosa in mice. A–C: Comparison of serum concentrations of triglycerides (TGs), total cholesterol, and free fatty acids after vehicle or cytarabine administration in mice (day 4). D–F: Total fecal lipid content, the average fecal lipid content per stool, and total number of excreted stools during the experimental period. G: Whole gastrointestinal motility using carmine red dye transit time after vehicle or cytarabine administration (day 3). H: Distribution of fluorescence signal intensity 2 h after FITC-dextran (70 kDa) gavage along the length of the gut (day 4). I: Mucosal concentration of TGs normalized by protein concentration of the duodenum and jejunum (Jej) after vehicle or cytarabine administration in mice (day 4). ∗*P* < 0.05, ∗∗*P* < 0.01, and ∗∗∗*P* < 0.001. Data in (A–G and I) represented as mean ± SEM were analyzed using one-way ANOVA followed by Tukey’s post hoc test, and data in (H) was analyzed using two-way ANOVA followed by Bonferroni’s post hoc test. *n* = 5–6 mice/group. CON versus CYT, ∗; CON versus PF, #; CYT versus PF, §. CON, control group treated with vehicle; CYT, group treated with cytarabine; PF, group pair-fed with the CYT group and treated with vehicle.

Given the lower fat mass and the higher RQ of the CYT group than those of the PF group, we hypothesized that CYT administration affected lipid metabolism in mice. Therefore, we compared the lipid content in the serum, feces, and mucosa of the mice from the three experimental groups. The serum TG levels were significantly lower in the CYT group than in the CON and PF groups, despite the consumption of energy intake being comparable to that of the PF group, whereas it was significantly higher in the PF group than in the CON group (Fig. 3A). Furthermore, the differences in the serum total cholesterol levels were insignificant among the three groups (Fig. 3B), whereas the serum-free fatty acid levels were lower in the CYT group than in the PF group (Fig. 3C). Therefore, the deficiency of serum lipids might contribute to the low utilization of lipid in the CYT group than in the PF group.

To investigate whether abnormal lipid homeostasis in the CYT group was associated with severe liver damage given that CYT is metabolized primarily in the liver (35, 36), we compared the levels of serum ALT and AST, biomarkers for liver damage, among the groups. The liver weight per body weight, serum ALT levels, and the ratio of AST and ALT were comparable among the three groups, and serum AST levels were increased in the CYT group compared to the CON group but comparable to the PF group (Fig. S2A–E). The results suggested no severe liver damage in the CYT group (37).

To identify the ability of intestinal lipid absorption in mice after CYT administration, we compared the total amount of fecal lipids among the three groups. The total amount of excreted fecal lipid was similar between the CYT and PF groups (Fig. 3D). However, the lipid content per stool was significantly higher in the CYT group than in the PF group (Fig. 3E), and the total fecal counts were lower in the CYT group (Fig. 3F). Next, we measured carmine red dye transit time on day 3 to assess the effect of CYT on whole intestinal transit. The CYT group showed delayed migration of carmine red dye compared to the CON and PF groups, while the PF group showed a faster transit than the CON and CYT groups (Fig. 3G). We also assessed the luminal transit of 70 kDa FITC-dextran on day 4 to determine the spatial distribution of intestinal transit. FITC-dextran was in the jejunum (segment number 4–5) in the CYT group 2 h after administration, while it was found in the cecum and colon (segment number 10–11) in the CON and PF groups (Fig. 3H). Consistently, the intestinal weight relative to length was higher in the CYT group than in the PF group (Fig. S2F), indicating that the luminal contents stayed in the small intestine for a longer period in the CYT group than in the other groups.

We further hypothesized that the lipids might be retained within intestinal mucosa of the CYT mice and compared the mucosal lipid levels in the duodenum and jejunum among the different groups. The levels of jejunum mucosal TGs, total cholesterol, and free fatty acids were significantly higher in the CYT group than those of the CON and PF groups (Figs. 3I and S2G, H). Thus, CYT treatment might reduce the ability of the enterocytes to transport lipids for circulation in the small intestine of mice after dietary fat intake.

### CYT reduces lipid absorption accompanied by lipid retention within the small intestine of mice

**Fig. 4:**
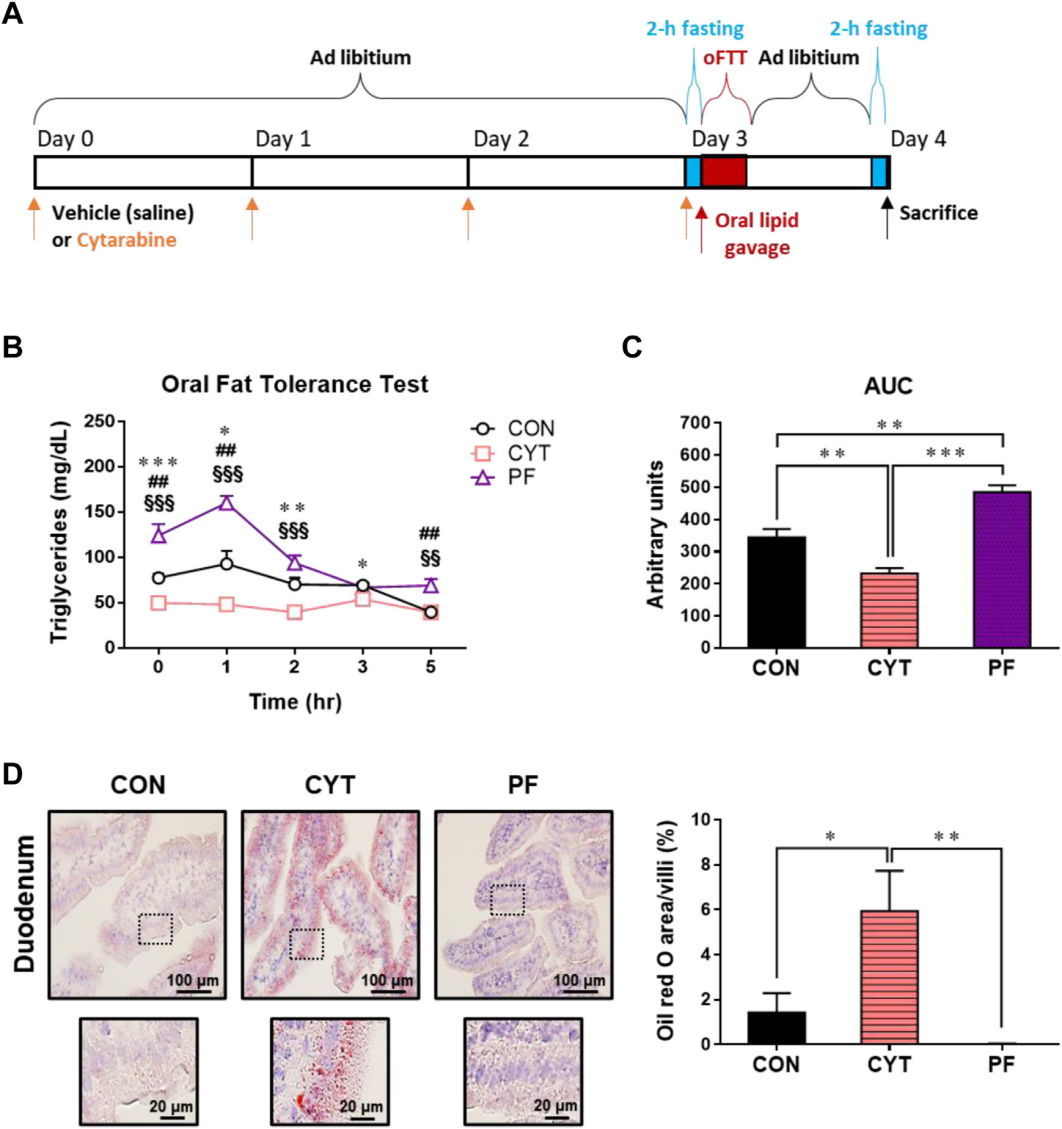
Cytarabine inhibits lipid absorption accompanied by lipid accumulation within the small intestinal mucosa in mice. A: Schematic representation of the experimental timeline and dosing schedule. B: Time-dependent changes in serum levels of triglycerides (TGs) after an oral olive oil loading (day 3). C: Area under the curve of (B). D: Representative images and comparison of neutral lipids of the small intestine stained with oil red O (ORO) post oral lipid loading (day 4). Each ORO-stained neutral lipid is indicated as arbitrary units of 3 villi/mouse (*n* = 6 mice/group). Thickness of tissues was 10 μm. Magnification, 40×. Scale bars, 100 μm. Enlarged panel; scale bars, 20 μm. ∗*P* < 0.05, ∗∗*P* < 0.01, and ∗∗∗*P* < 0.001. Data in (B–D) are represented as mean ± SEM. Data in (B) were analyzed using two-way ANOVA followed by Bonferroni’s post hoc test, and data in (C, D) were analyzed using one-way ANOVA followed by Tukey’s post hoc test. *n* = 6 mice/group. All groups were orally administered lipid on day 3 and sacrificed on day 4. Significance: CON versus CYT, ∗; CON versus PF, #; CYT versus PF, §. CON, control group treated with vehicle; CYT, group treated with cytarabine; PF, group pair-fed with the CYT group and treated with vehicle.

We directly compared the changes in the serum TG levels after an oral lipid loading among the three treatment groups (Fig. 4A). The serum TG levels were significantly lower in the CYT group during the course of experiment after the lipid loading than in the CON and PF groups (Fig. 4B). The AUC for the serum TG levels during the oFTT was significantly lower in the CYT group than in the CON and PF groups, indicating reduced lipid absorption in the CYT group, whereas the AUC was significantly higher in the PF group than in the CON group (Fig. 4C). The serum TG levels decreased more rapidly in the PF group than in the CON and CYT groups, whereas the same were unaltered during the oFTT in the CYT group, indicating enhanced utilization of TGs in the PF group.

To confirm the increase in lipid retention within the small intestinal mucosa post CYT administration, ORO staining of the sections of the small intestine was performed 24 h after the oral lipid loading in mice. The RO+ area was more frequently observed in the mucosa of the duodenum of the CYT group than in that of the CON and PF groups (Fig. 4D). In another study without oral lipid administration, ORO staining showed similar patterns in mice on a chow diet (Fig. S3A). These findings suggested that CYT administration might persistently disturb the process of lipid metabolism, including lipid absorption and utilization in mice.

To identify whether CYT-induced decrease in the intestinal lipid absorption was associated with any damage in the intestinal mucosa, we compared various parameters related to intestinal damage, such as the expression of genes associated with macrophage recruitment and proinflammation, as well as the intestinal villus length, intestinal length, crypt depth, LGR5-positive cells, and permeability using a 4 kDa FITC-dextran permeability assay, among the different treatment groups. The expression of the proinflammatory genes, including *Il1b*, *Tnf*, *Ccl2*, and *Il6*, the serum FITC-dextran levels and after oral FITC-dextran loading, and the intestinal length were not different among the three groups (Fig. S4A, D and E). Moreover, the intestinal villus length was not different between the CON and CYT groups (Fig. S4C). The expression of macrophage recruitment-related genes, including *Adgre1* and *Itgax*, was higher in the CYT group than that in the PF group (Fig. S4B). The number of LGR5-positive cells, a marker for intestinal stem cell, was significantly lower in the CYT group than in the CON and PF groups, while the crypt depth was significantly longer in the CYT group than in the CON group and comparable to the PF group (38, 39) (Fig. S4F–H). Thus, reduced lipid absorption by CYT was unlikely to be associated with overt intestinal damage except for the decreased LGR5-positive cells, which requires further investigation (40, 41, 42).

### CYT increases the size of chylomicrons within trans-Golgi network in the small intestine of mice.

To identify the underlying mechanism of lipid retention within the small intestinal mucosa after CYT administration, the ultrastructure of the duodenal epithelial cells was compared using transmission electron microscopy (TEM) among the different treatment groups.

**Fig. 5:**
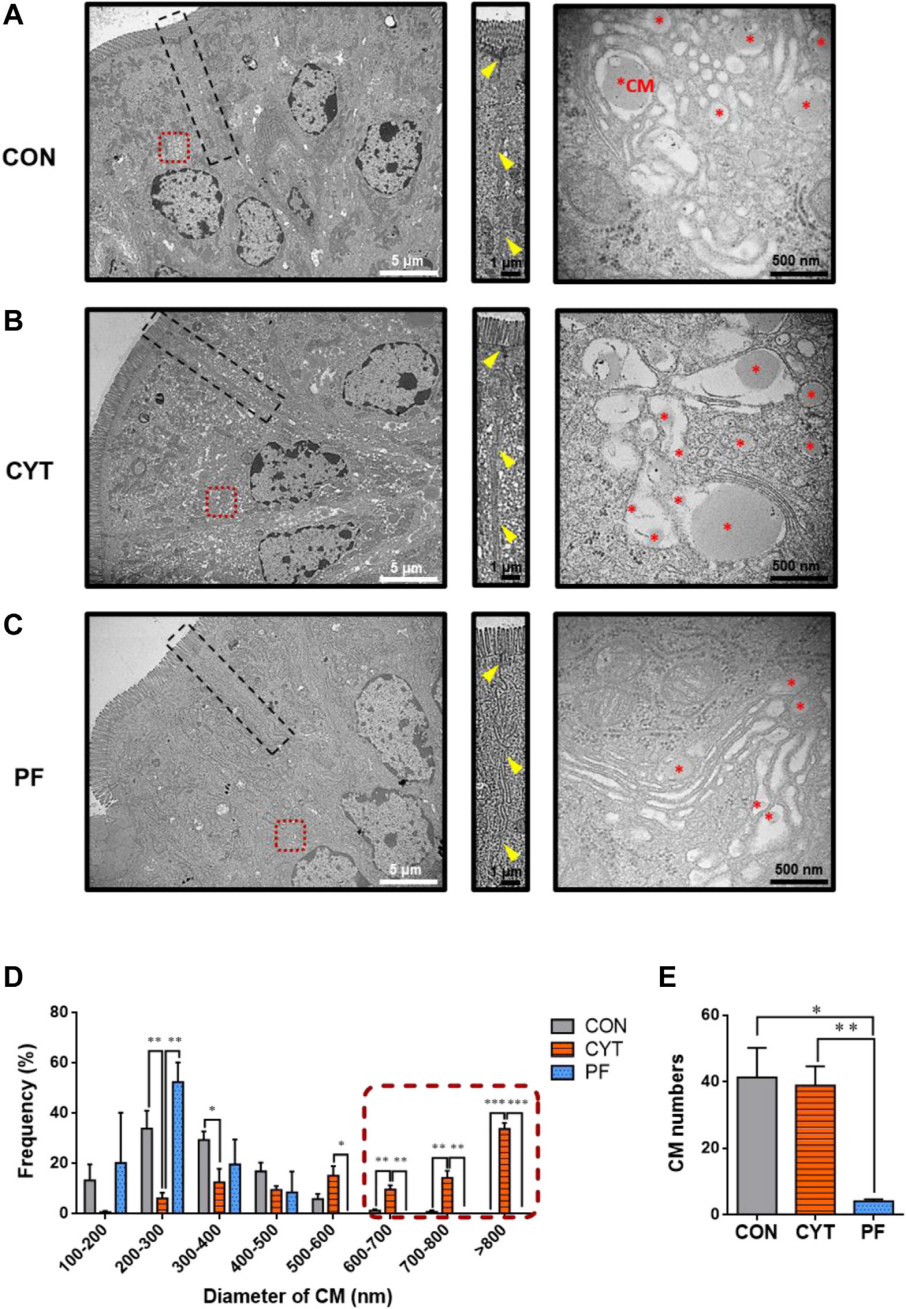
Cytarabine increases the size of chylomicrons in the duodenal enterocytes of mice. A–C: Representative transmission electron micrograph (TEM) of duodenal enterocytes of mice treated with vehicle or cytarabine (day 4). Black-dashed box, cell-to-cell junction. Red-dashed box, trans-Golgi network (TGN). Left panel magnification and scale bars, 6000× and 5 μm. Center panels represent magnified views of black-dashed box of (A–C). Yellow arrowhead, tight junction. Right panels represent magnified views of red-dashed box of (A–C). Scale bars, 1 μm. Chylomicrons (CMs) in TGN of enterocytes in mice after vehicle or cytarabine administration (day 4). Red asterisk, CM. Right panel magnification and scale bars, 60,000× and 500 nm. D: Summarization of CMs size within the TGN of each group. Dashed box indicates the frequencies of abnormally large sizes of CM within TGN (diameter > 600 nm). *n* = 3–5 enterocytes/group. E: The comparison of the number of CMs within the TGN. *n* = 3–5 enterocytes/group. ∗*P* < 0.05, ∗∗*P* < 0.01, and ∗∗∗*P* < 0.001. Data in (D) are represented as mean ± SEM and analyzed using two-way ANOVA followed by Bonferroni’s post hoc test, and data in (E) were analyzed using one-way ANOVA followed by Tukey’s post hoc test. CON, control group treated with vehicle; CYT, group treated with cytarabine; PF, group pair-fed with the CYT group and treated with vehicle.

The tight junctions of the enterocytes were intact in all the experimental groups, which was consistent with the results obtained for intestinal damage (Fig. 5A–C, center panels). The immature pre-chylomicrons (CMs) were more frequently observed within the endoplasmic reticulum and Golgi apparatus in the CYT group than in the CON and PF groups (Fig. 5A–C). Abnormally large CM sizes were observed in the distended cisternae of trans-Golgi network (TGN) of the CYT group (Fig. 5B, right panel) compared to that in the CON and PF groups (Fig. 5A, C, right panel). The average diameter of CM was determined to be within the normal range in the CON and PF groups, which ranges from 75 nm to a maximum of 600 nm (43, 44). However, the average diameter of CM in the distended cisternae of TGN was prominently increased (>600 nm) in the CYT group compared to that in other groups (Fig. 5D, red-dashed box). Consistent with the large sizes of CM in the CYT group, the ratio of mucosal ApoB-48 and TG concentration was significantly decreased in the CYT group compared to the CON and PF groups (Fig. S5A–C). The average number of CM was significantly higher in the CON and CYT groups than in the PF group (Fig. 5E).

### CYT accumulates CMs within the intercellular space of enterocytes in mice

**Fig. 6:**
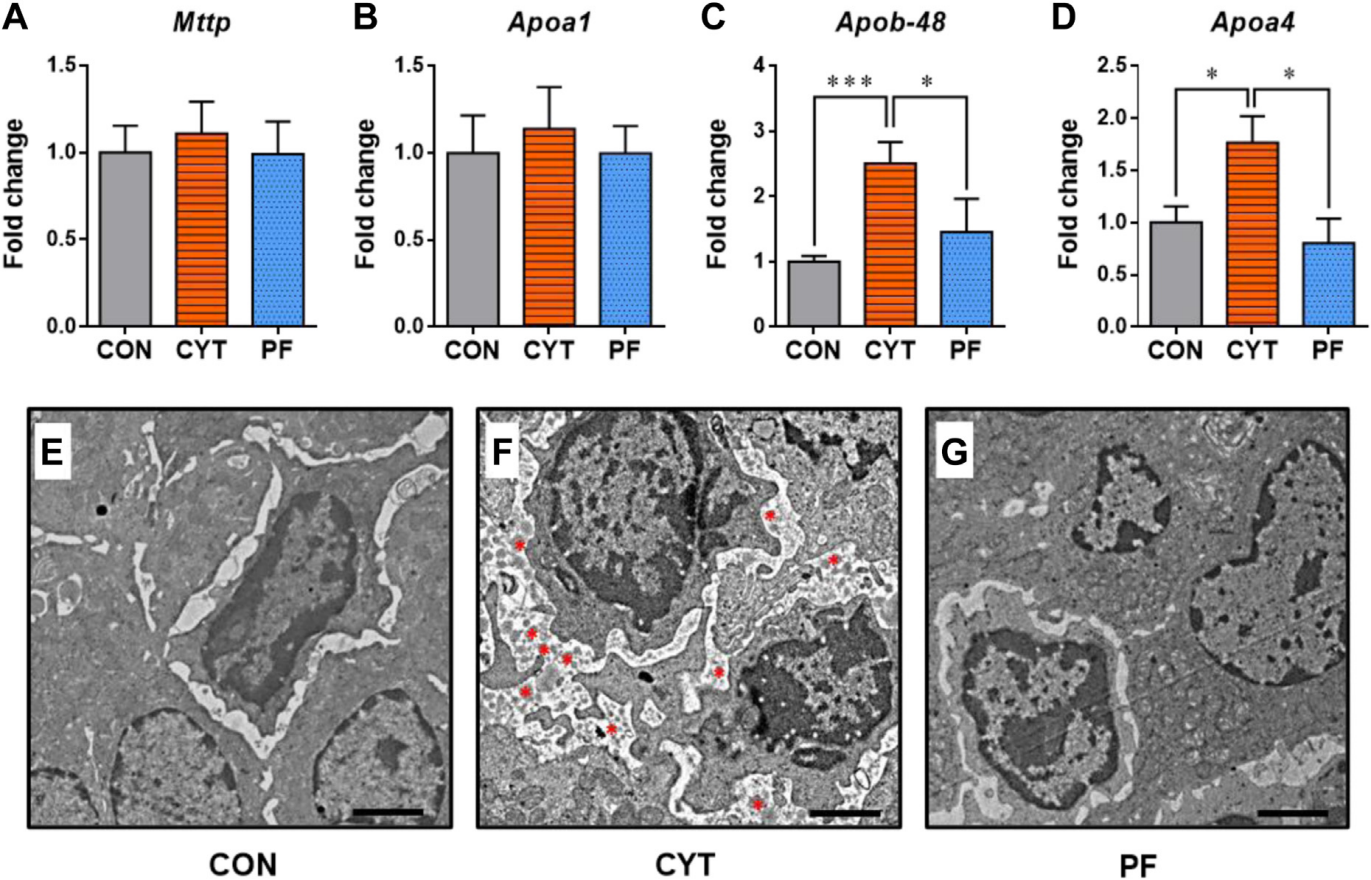
Cytarabine accumulates secreted chylomicrons within intercellular space of enterocytes in the small intestine of mice. A–D: Comparison of relative mRNA levels of lipid metabolism-associated genes of duodenal mucosa among the indicated groups after vehicle or cytarabine administration (day 4). E–G: Representative transverse-sectional TEM of duodenal enterocytes in mice after vehicle or cytarabine administration (day 4). Secreted chylomicrons are indicated by red asterisk. Magnification, 15,000×. Scale bar, 2 μm. ∗*P* < 0.05 and ∗∗∗*P* < 0.001. Data in (A–D) are represented as mean ± SEM and analyzed using one-way ANOVA followed by Tukey’s post hoc test. *n* = 5–6 mice/group. CON, control group treated with vehicle; CYT, group treated with cytarabine; PF, group pair-fed with the CYT group and treated with vehicle.

To investigate the process of lipid transport via the small intestine, the expression of genes related to CM synthesis, including *Mttp*, microsomal TG transfer protein that mediates packaging of lipids, and *Apoa1*, *Apob48*, and *Apoa4* involved in packaging and maturating pre-CM transport vesicle from endoplasmic reticulum to Golgi, in the duodenal mucosa was compared among the treatment groups (27, 45, 46). The results revealed that the expression of *Mttp* was not significantly different among the groups. However, the expression of *Apob48* and *Apoa4* was significantly higher by 2.5-fold or 1.8-fold in the CYT group than in the CON and PF groups (Fig. 6A–D). Additionally, we observed an increase in the gene expression of *Apobec-1*, an enzyme responsible for posttranscriptional RNA modification of *Apob48*, in the CYT group compared to the PF group (47) (Fig. S5D). These findings suggest the possibility of increased demand for CM synthesis in the CYT group compared to that in the other treatment groups.

Furthermore, lipid particles were more frequently observed within the intercellular space between the duodenal enterocytes in the CYT group than in other groups, suggesting the involvement of another process for lipid transportation, including that mediated by lacteal, with the CYT-induced lipid retention (48, 49) (Fig. 6E–G).

### CYT increases zipper-like lacteal junctions in the small intestine of mice

**Fig. 7:**
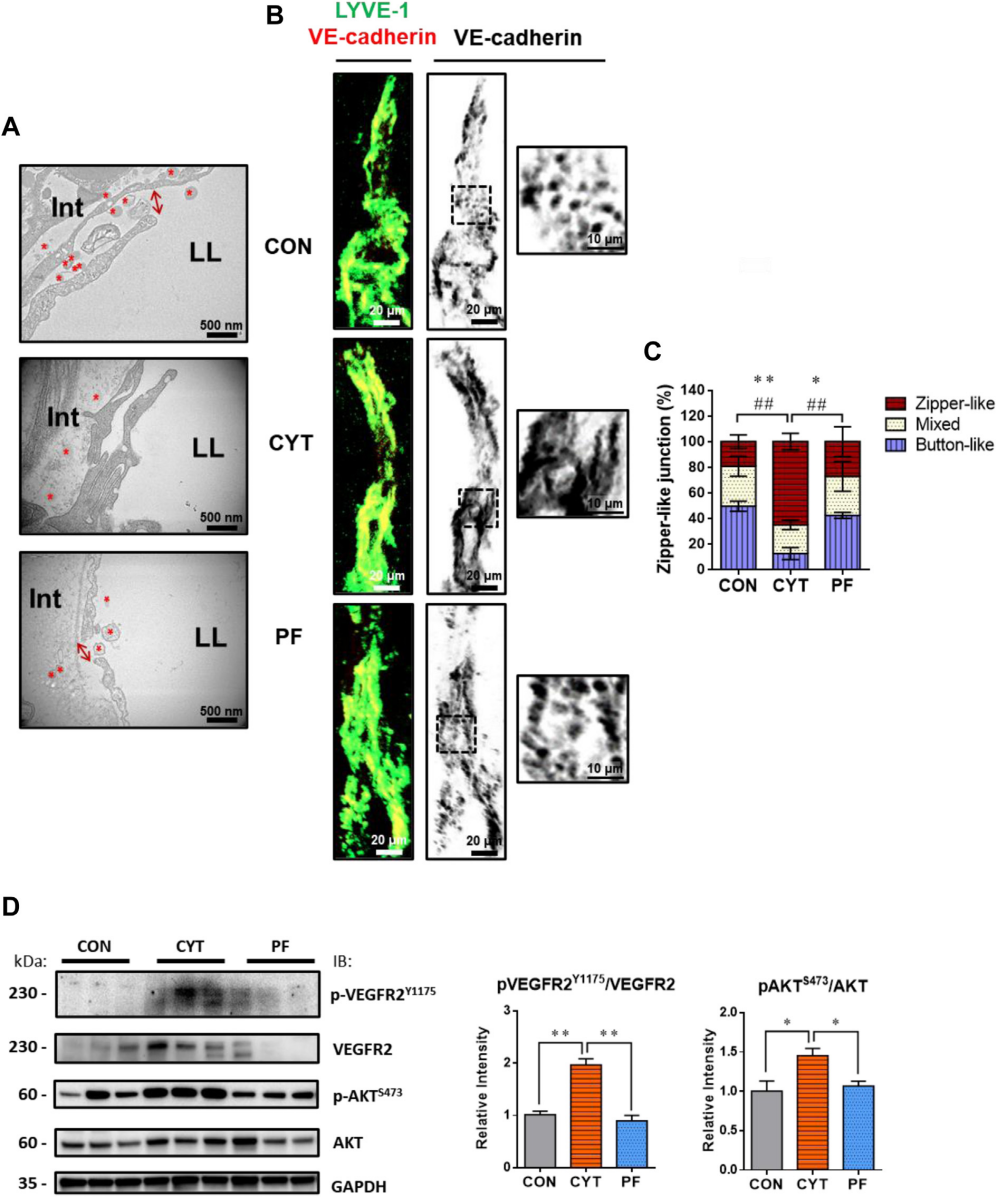
Cytarabine increases zipper-like lacteal junctions in the small intestinal villi of mice. A: Representative TEM of duodenal enterocytes in mice after vehicle or cytarabine administration (day 4). Magnification, 50,000×. Scale bars, 500 nm. B, C: Representative micrographs and comparison of VE-cadherin^+^ junctions of jejunal lymphatic endothelial cell (LEC) of LYVE1^+^ lacteals in mice after vehicle or cytarabine administration (day 4). Scale bars, 20 μm. Black-dashed box is magnified in the right panel. Scale bars, 10 μm. Five to six villi per mouse, *n* = 3 mice/group. D: Western blot analysis and densitometric quantification of VEGFR2, p-VEGFR2^Y1175^, AKT, and p-AKT^S473^ expression in jejunum mucosal protein of mice after vehicle or cytarabine administration (day 4). (*n* = 3 per condition). GAPDH protein is the loading control. ∗*P* < 0.05 and ∗∗*P* < 0.01. Data in (C) represented as mean (SD) and (D) mean ± SEM were analyzed using one-way ANOVA followed by Tukey’s post hoc test. Significance, zipper-like junction, ∗; button-like junction, #. Red asterisk, CM. CON, control group treated with vehicle; CYT, group treated with cytarabine; Int, interstitium of lamina propria; LL, lacteal lumen; kDa, kilodalton; PF group pair-fed with the CYT group and treated with vehicle.

The junctional status of LEC is critical for CM uptake into the lymphatic circulation (30, 50). To investigate whether CYT administration inhibits intestinal lipid absorption by changing the junctions of LEC, the microstructure of junctions of LECs among the different treatment groups were compared by performing morphological studies with TEM imaging and double immunostaining. The overlapped and closed junctions of LEC (zipper-like junctions) were frequently observed in the CYT group with the presence of CMs within the interstitium of lamina propria, whereas they were barely detected in the lacteal lumen (51) (Fig. 7A, CYT). On the contrary, paracellularly opened lacteal junctions (button-like junctions) were observed in the CON and PF groups, whereas the CMs were frequently detected in the lacteal lumen of both the groups (Fig. 7A, CON and PF). Furthermore, the immunostaining of lacteal junctions with VE-cadherin displayed mostly discontinuous button-like junctions in the CON and PF groups. However, the proportion of impermeable zipper-like junctions was more in the CYT group than in the CON and PF groups by 3.4-fold and 2.4-fold (Fig. 7B, C). The activation of VEGFR2/AKT pathway regulates the morphological changes of VE-cadherin junctions in LECs, thereby contributing to the increased proportion of zipper-like junctions (49, 52). Consistently, the phosphorylated form of VEGFR2 at tyrosine-1173 (Y1173) and that of AKT at serine 473 (S473) were significantly increased in the CYT group compared to the CON and PF groups (Fig. 7D).

**Fig. 8:**
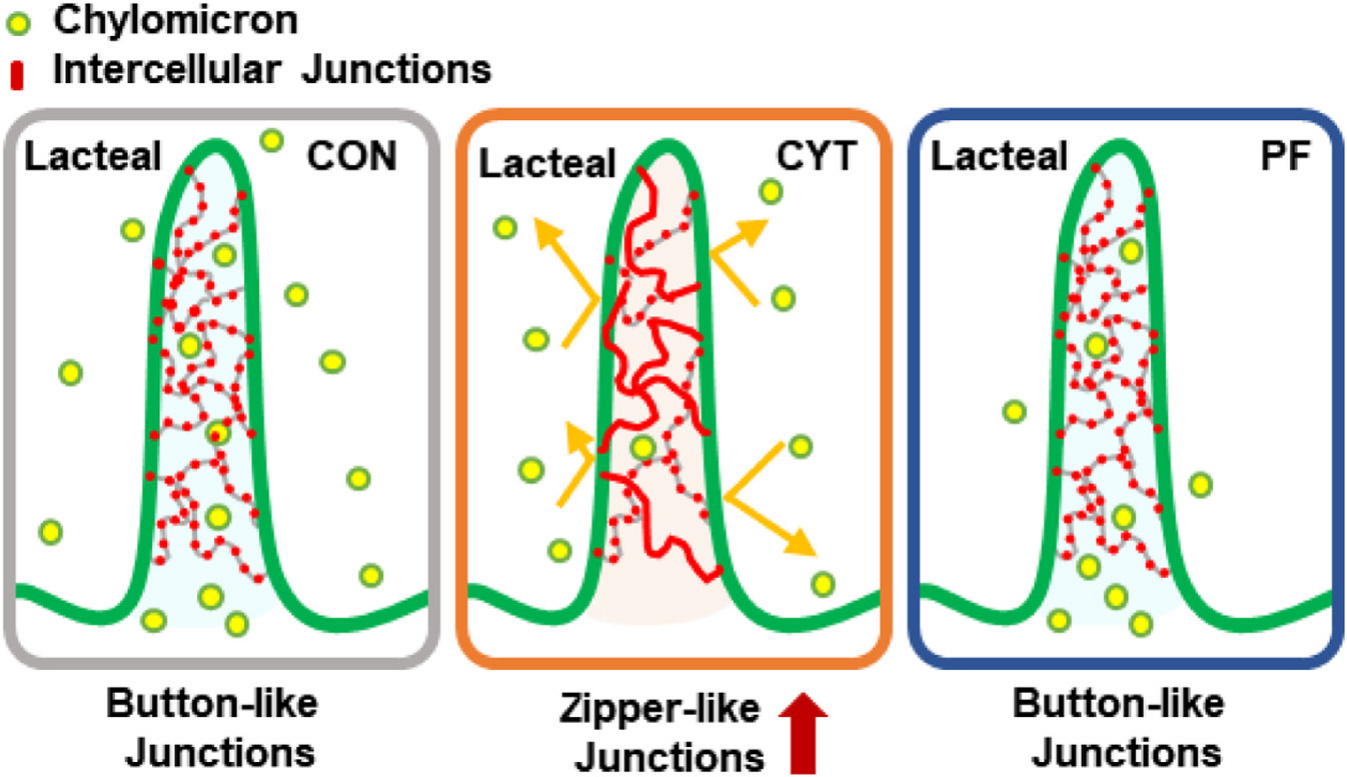
Cytarabine reduces lipid absorption via increasing zipper-like lacteal junctions of small intestine in mice. Schematic figure presenting the mechanism underlying cytarabine-induced decrease in lipid uptake. Cytarabine increases zipper-like junctions of the lacteal within the small intestine and subsequently inhibits intestinal lipid uptake in mice.

Overall, the increased proportion of lacteal in the zipper-like junctions might contribute to the suppression of lipid transport into lymphatic circulation and subsequent decrease in lipid absorption in the CYT group compared to that in the CON and PF groups (Fig. 8A).

## Discussion

The aim of this study was to identify the underlying mechanisms of chemotherapy-induced energy imbalance in the CYT-induced cachexia mouse model. In this study, we established the cachexia mouse model and determined the effects of CYT on the regulation of energy balance with PF mice serving as control. Additionally, we provided evidence that intestinal lipid malabsorption greatly contributed to the CYT-induced cachexia that is independent of the CYT-induced anorexia. Furthermore, we demonstrated that the CYT-induced intestinal lipid malabsorption might be attributable to intestinal lipid retention via the increased proportions of zipper-like junctions of LEC in the villi of small intestine. Therefore, to the best of our knowledge, this is the first study that emphasizes the critical role of zipper-like junctions of LEC within the small intestine in the exacerbation of the CYT-induced cachexia that is independent of the anorexia.

We elucidated that CYT administration led to weight loss with a marked decrease in metabolic efficiency. Moreover, the CYT group exhibited greater weight loss despite lower EE than that exhibited by the PF group, whereas both the groups consumed the same amount of food. Consistently, we observed reduced *Ucp1* expression in both the BAT and eWAT of the mice belonging to the CYT group compared to those of the PF group, indicating decreased thermogenesis. Additionally, this was accompanied by elevated loss of muscle and fat mass and lower grip strength in the CYT group than those in the PF group. Thus, CYT administration induced cachexia that was different from the anorexia-induced weight loss, implicating the existence of a factor capable of worsening the cachexia independent of anorexia.

Our results demonstrated that the CYT group consumed less lipid than the PF group although both the CYT and PF groups demonstrated comparable, albeit smaller amount of dietary intake than the CON group. This was corroborated by the lower serum fatty acid levels and lipolytic gene expression in adipose tissues of the mice belonging to the CYT group than those of the PF group (Figs. 3C and S5E, F). Enhanced lipolysis is considered one of the main factors for the development of cancer cachexia (43, 44, 53, 54, 55). However, chemotherapeutic agents cause weight and fat losses by differently affecting lipid metabolism in WAT. Cisplatin or combined treatment with irinotecan (CPT-11) and 5-fluorouracil increases WAT lipolysis via the activation of fatty acid oxidation in rodent models (45, 46, 56, 57, 58), whereas doxorubicin inhibits both lipolysis and lipogenesis in WAT (48, 59). Therefore, given the higher RQ and lower lipolytic gene expression in WAT, despite the lower fat mass in the CYT group than that in the PF group, the low serum TG levels post CYT administration might be attributable to the lower intestinal lipid absorption in the CYT group than in the PF group.

CYT administration might indirectly reduce skeletal muscle function and mass in mice. In an in vitro study using C2C12 myoblasts, CYT treatment affected contractile ability and sarcomere organization, indicating a lack of its direct effect on muscle function and mass (12, 16). However, we observed a significant decrease in the skeletal muscle mass and grip strength in mice after CYT administration. Thus, the in vivo effects of CYT on muscle might be indirect and associated with other factors such as the alterations in metabolism, although further investigation is needed.

Next, we investigated the effect of CYT treatment on intestinal lipid absorption in mice. The lipid content in serum was significantly lower in the CYT group than in the PF group, while the total amount of excreted fecal lipid was comparable between the CYT and PF groups. These results prompted us to compare the lipid contents in the intestinal mucosa among the treatment groups. The lipid contents were significantly increased only in the intestinal mucosa of the CYT group compared to those of the other groups, indicating CYT-induced lipid retention in the mucosa. Furthermore, we confirmed that CYT treatment suppressed lipid absorption post oral lipid loading with the potentiated effect of CYT on lipid retention in mice (Figs. 4 and S6).

Our results revealed that lipid retention in the intestinal mucosa was not associated with the changes in structure and permeability of mucosa. We observed intact tight junctions and no significant differences in the serum FITC-dextran levels indicative of unaffected intestinal permeability between the cells in the intestinal mucosa of all the treatment groups. Additionally, no significant differences in the expression of proinflammatory genes, except for the macrophage recruitment-associated genes, were detected indicating no evident signs of leaky gut post-CYT administration (49, 60). This is different from the findings of studies on other chemotherapeutic agents like doxorubicin or 5-fluorouracil, which cause severe mucositis and increased intestinal permeability (50, 51, 61, 62). It is noteworthy that the number of LGR5-positive cells decreased post-CYT administration, while morphometric parameters, such as the intestinal villi length and crypt depth, were similar between the CYT and PF groups. This might affect the function of lipid absorption in the intestinal epithelium despite the existence of the heterogenous pools of stem cells (38, 39, 63). Further investigation is needed to determine the effect of CYT-induced alterations in stemness on lipid metabolism. Thus, the results of the present study emphasize that the process governing intestinal transport of lipids into lymphatic circulation holds greater significance in the CYT-induced intestinal lipid malabsorption than the repercussions associated with leaky gut or intestinal damage.

We further investigated the ultrastructural changes in the enterocytes of small intestine to identify the underlying mechanisms of the CYT-induced lipid retention within the intestinal mucosa of mice. We observed an increased proportion of zipper-like junctions of LEC in the CYT group compared to that in the other groups, as per TEM and immunohistochemistry analyses of LEC and VE-cadherin expression, as well as activation of VEGFR2/AKT pathway, which is a key signaling pathway for lacteal junction zippering (49, 52) (Fig. 7). CMs enter the lymphatic circulation in a size-exclusive manner due to the high proportion of button-like junctions of LEC within the small intestinal villi. Recent studies have shown that the systemic uptake of CM is inhibited by the occurrence of zipper-like lacteal junctions (29, 30, 48, 49, 50, 64). Given the accumulation of the large sizes of CM in the cisternae of trans-Golgi apparatus of the enterocytes with abnormal CM metabolism and the detection of secreted CM particles in the intercellular space of enterocytes in the CYT group, the increased proportion of zipper-like junctions in the CYT group might contribute to the suppression of CM transport via the junctions into circulation. Therefore, the results suggest the important role of button-to-zipper transformation in the CYT-induced lipid retention (Fig. 7B).

The factors regulating the size of CMs during the addition of lipids are uncertain. ApoA-IV is incorporated into nascent CMs and is suggested to have an important role in the regulation of CM size in a way of facilitating the packaging of additional lipids into the core to produce a larger particle (45, 65). Indeed, large sizes of CM were detected in transgenic mice overexpressing Apo-AIV (66). Therefore, the increased expression of *Apoa4* in the CYT group might contribute to the production of larger CMs observed within the enterocytes.

The regulatory system linking the button-to-zipper transformation and the CM synthesis has not yet been studied. Mucosal TG accumulation and reduced CM uptake were observed in *Lats1/2iΔPβC* or *Nrp1;Flt1ECko* mice that displayed increased zipper junctions (48, 49). Given that the increased CM production and the increased zipper-like junction in the CYT group, there might exist independent regulatory systems for them, which requires further investigation.

In this study, we observed a decrease in intestinal transit induced by CYT administration, which might affect the process of lipid absorption. The correlation between intestinal transit and lipid absorption is dependent on various conditions (67, 68, 69), and the underlying mechanism remains unclear. Notably, we observed larger and more CMs in the enterocyte and intercellular space but not in the lacteal lumen when the luminal contents stayed longer in the jejunum, which is one of the primary sites for lipid absorption (45). These findings might emphasize the important role of the zipper-like junctions in the lipid transit from the lumen to circulation in the CYT group.

In previous studies, it has been reported that chemotherapeutic agents, such as paclitaxel and platinum-based drugs, disrupt lipid homeostasis both in human and mice (62, 70). Our study is the first to highlight the role of lacteal junction in lipid malabsorption in a chemotherapy-induced cachexia mouse model. It is important to note that cancer cachexia and chemotherapy-induced cachexia have different metabolic perturbations, including intestinal barrier dysfunction, abnormal lipid metabolism, and inflammation, which vary depending on the type of chemotherapy or cancer (71, 72, 73, 74, 75).

Delineating the underlying mechanisms of chemotherapy-induced cachexia is essential to develop a way to maintain energy balance for successful cancer treatment (2, 14, 19). In this study, CYT treatment worsened cachexia in ways that were independent from the anorexia-induced weight loss. Moreover, it resulted in dysfunctional lipid absorption with no evident signs of leaky gut, thereby contributing to the development of CYT-induced anorexia-independent cachexia in mice. Furthermore, the exacerbation of CYT-induced cachexia might be due to the increased proportion of zipper-like junctions of lacteal within the small intestine and subsequent intestinal lipid retention. Consequently, our findings provide evidence for a critical role of lacteal in the CYT-induced cachexia. Overall, our findings suggest that modulating the lacteal might be an important mechanism for maintaining energy balance during the CYT treatment, which can be potentially explored to develop a novel strategy for sustainable cancer treatment.

## Data availability

All described data are contained within the article.

## Supplemental data

This article contains supplemental data.

## Conflict of interest

The authors declare no conflict of interests with this article contents.
