# Supplementary material for: Cytarabine induces cachexia with lipid malabsorption via zippering the junctions of lacteal in murine small intestine
Source: J Lipid Res. 2023 May 16;64(6):100387. doi: 10.1016/j.jlr.2023.100387 (PMC10323926; doi:10.1016/j.jlr.2023.100387)
Supplement: Supplemental Figures and Tables [file mmc1.docx]

*Supporting Information*

***Cytarabine induces anorexia-independent cachexia via zipper-like junctions of lacteal in murine small intestine***

Authors

Mi-Rae Park ^a, b^, Hye-Jin Lee ^a^, Hye-Min Jang ^a^, Nam Hoon Kim ^c^, Jun-Seok Lee ^a^, Yong Taek Jeong ^a, b^, Inho Kim ^d^ Sang-Hyun Choi ^a^, Kwan Sik Seo ^e^, Dong-Hoon Kim ^a, b^

^a^ Department of Pharmacology, Korea University College of Medicine, Seoul 02841, Republic of Korea

^b^ Department of Biomedical Sciences, Korea University College of Medicine, Seoul 02841, Republic of Korea

^c^ Division of Endocrinology and Metabolism, Department of Internal Medicine, Korea University College of Medicine, Seoul 02841, Republic of Korea

^d^ Department of Internal Medicine, Seoul National University College of Medicine, Seoul National University Hospital, Seoul 03080, Republic of Korea.

^e^ Department of Rehabilitation Medicine, Seoul National University Hospital, Seoul 03080, Republic of Korea .

*Supplementary Figures*

Fig. S1 Comparison of metabolic glucose status, brown adipose tissue mass, and *Ucp1* expression in mouse adipose tissues. (a-c) Comparison of the 2-h fasting blood glucose and glycogen content in skeletal muscle and liver after vehicle or cytarabine treatment (day 4). (d) Comparison of the brown adipose tissues (BAT) mass after vehicle or cytarabine treatment (day 4). (e) Comparison of uncoupling protein 1 (*UCP1*) mRNA expression in the BAT, epididymal white adipose tissue (eWAT), and inguinal white adipose tissue (iWAT) after vehicle or cytarabine administration (day 4). mRNA expression is presented as fold change relative to that in the CON group.

**P* < 0.05, ***P* < 0.01, and ****P* < 0.001. Data in (a-e) are represented as mean ± SEM and analyzed using one-way ANOVA followed by Tukey’s post hoc test. *n* = 5-6 mice/group.

Fig. S2 Effect of cytarabine on the liver damage indicators and the contents of total cholesterol and free fatty acids in the small intestinal mucosa in mice. (a, b) Representative images of liver, liver weight, and liver-to-body weight ratio after vehicle or cytarabine administration in mice (day 4). (c-e) Serum content of alanine aminotransferase (ALT) and aspartate aminotransferase (AST) and a ratio of AST/ALT from (c, d) after vehicle or cytarabine treatment (day 4). (f) Intestinal weight relative to length ratio after vehicle or cytarabine treatment (day 4). (g, h) Mucosa concentration of total cholesterol and free fatty acid after vehicle or cytarabine administration in mice (day 4).

**P* < 0.05, and ***P* < 0.01. Data in (b-h) are represented as mean ± SEM and analyzed using one-way ANOVA followed by Tukey’s post hoc test. *n* = 6 mice/group.


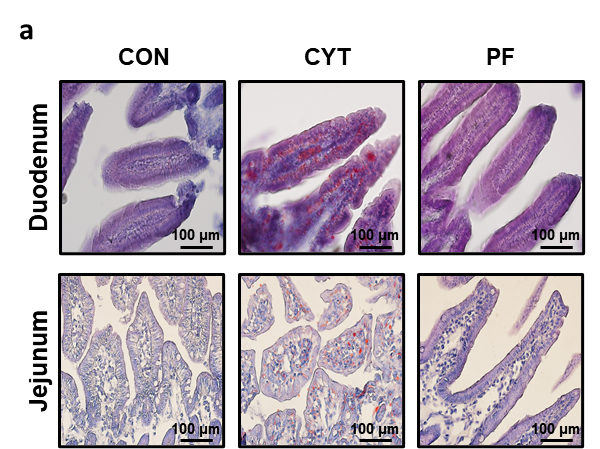


Fig. S3 CYT increases lipid accumulation in the small intestine under chow-fed condition. (a) Representative images and comparison of neutral lipids of the small intestine stained with Oil-red-O (ORO) after vehicle or CYT administration (day 4). Thickness of tissues was 30 μm in duodenum and 10 μm in jejunum. Scale bars, 100 μm. Magnification, 40×.


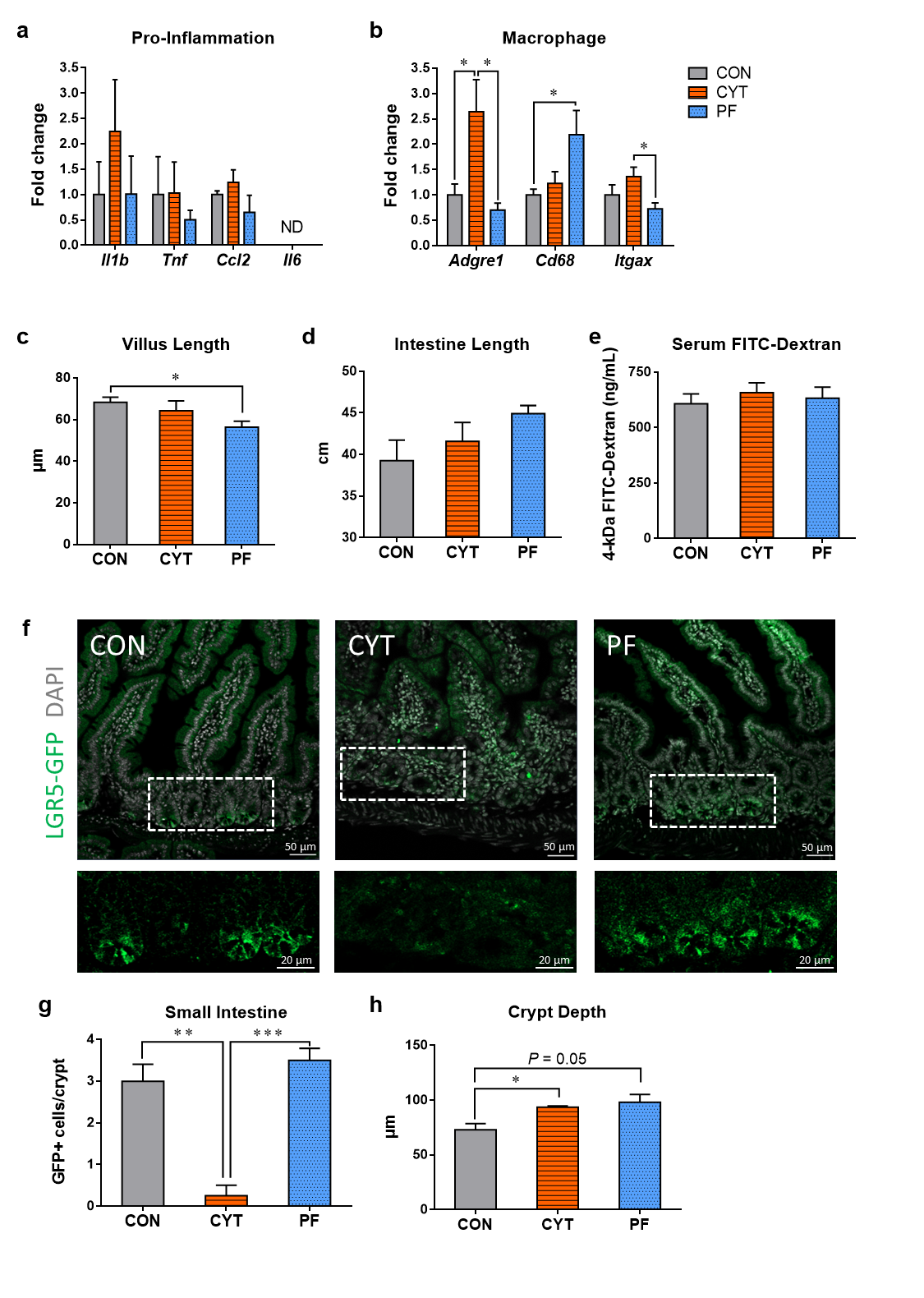


Fig. S4 Involvement of cytarabine with leaky gut in mice. (a, b) Relative mRNA expression level of pro-inflammatory and macrophage recruitment-associated markers in the duodenal mucosa of mice treated with vehicle or cytarabine (day 4). (c) Comparison of duodenal villus length after vehicle or cytarabine administration (day 4). (d) Comparison of whole intestine length after vehicle or cytarabine administration (day 4). (e) Comparison of serum FITC-dextran concentration after oral FITC-dextran load (day 4). (f, g) Representative micrographs and comparison of GFP+ stem cells in *Lgr5-EGFP-IRES-creERT2* mice after vehicle or cytarabine administration (day 4). Scale bars, 50 μm. White dashed box is magnified in the below panel. Scale bars, 20 μm. (h) Comparison of crypt depth in *Lgr5-EGFP-IRES-creERT2* mice after vehicle or cytarabine administration (day 4).

**P* < 0.05, and ***P* < 0.01. Data in (a-e and g-h) are represented as mean ± SEM and analyzed using one-way ANOVA followed by Tukey’s post hoc test. *n* = 5-6 mice/group.


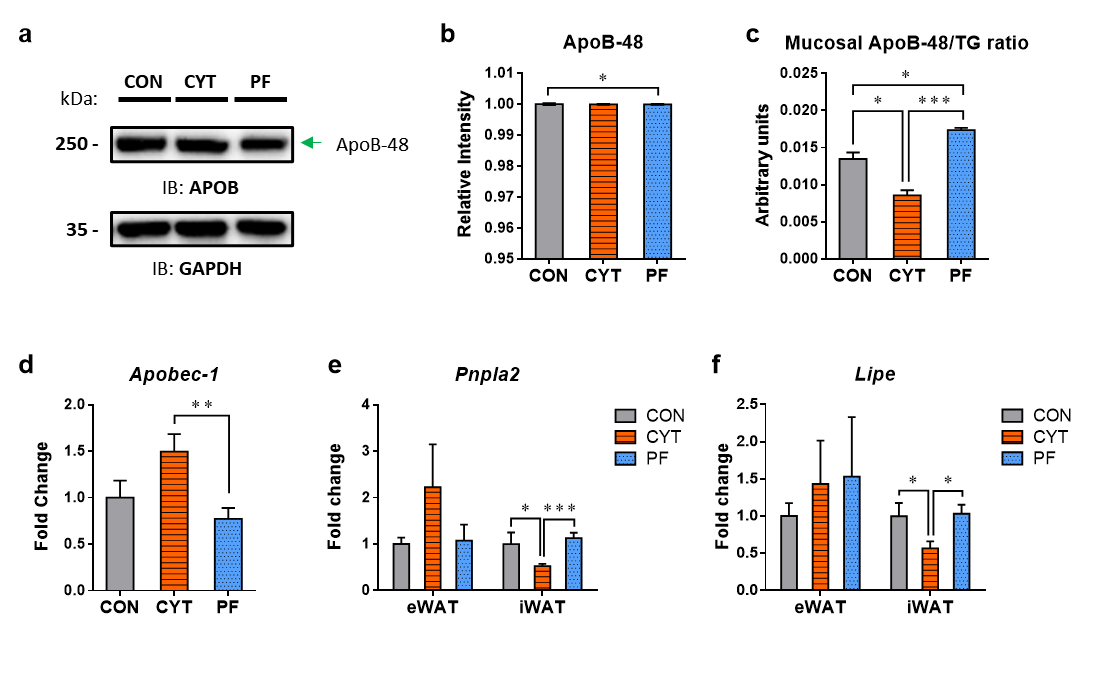


Fig. S5 Effects of cytarabine on CM production gene and protein expression in duodenal mucosa, and lipolytic gene expression in the WAT of mice. (a, b) Western blot analysis and densitometric quantification of ApoB-48 and GAPDH expression in duodenum mucosal protein of mice after vehicle or cytarabine administration (day 4). GAPDH protein is the loading control. kDa, kilodalton. (c) Mucosal TG content-to-ApoB-48 relative intensity ratio after vehicle or cytarabine administration in mice (day 4). (d) Relative mRNA expression of *Apobec-1* of duodenal mucosa among the indicated groups after vehicle or cytarabine administration (day 4). (e, f) Relative mRNA expression of lipolytic genes in epididymal white adipose tissue (eWAT) and inguinal white adipose tissue (iWAT) of mice treated with vehicle or cytarabine (day 4).

**P* < 0.05, ***P* < 0.01, and ****P* < 0.001. Data in (a-f) are represented as mean ± SEM and analyzed using one-way ANOVA followed by Tukey’s post hoc test. (b, c) *n* = 3 per condition. (d-f) *n* = 6 mice/group.

Fig. S6 Comparison of the levels of lipid accumulation in duodenum among the mice from the CON, CYT, and PF groups. (a) Comparison of neutral lipid of duodenum stained with Oil-red-O (ORO) after 2-hour fasting or oral lipid load (LL) after vehicle or cytarabine administration in mice (day 4). ORO area of each group was calculated as fold change relative to that of the CON group.

**P* < 0.05. Data is represented as mean ± SEM and analyzed using one-way ANOVA followed by Tukey’s post hoc test. *n* = 5-6/group. Significance; CON vs other groups.

*Supplementary Tables*

Table S1. TaqMan probe used for RNA quantification.

All genes were normalized with the housekeeping gene *Rpl32*

| Gene name | TaqMan probe |
| --- | --- |
| *Ucp1* | Mm01244861_m1 |
| *Pnpla2* | Mm00503040_m1 |
| *Ccl2* | Mm00441242_m1 |
| *Il6* | Mm00446190_m1 |
| *Apob* | Mm01545150_m1 |
| *Rpl32* | Mm02528467_g1 |

Table S2. Primer sequences used for RNA quantification.

All genes were normalized with the housekeeping gene *Rpl32*

| Gene name | Oligonucleotides |
| --- | --- |
| *Mttp* | Forward 5'-CCA GGG CTT TTG CCT TGA AC-3' |
|  | Reverse 5'-GAG GAC CTG TCC CAC AAT GG-3' |
| *Apoa4* | Forward 5'-GCG TGC AGG AGA AAC TCA AC-3' |
|  | Reverse 5'-GCT GGT CGA TTT TTG CGG AG-3' |
| *Apoa1* | Forward 5'-GAA CGA GTA CCA CAC CAG GG-3' |
|  | Reverse 5'-TGG CCT TGT CGA TCA CAC TC-3' |
| *Apobec-1* | Forward 5'-CCG AAC ACC AGA TGC TCC AT-3' |
|  | Reverse 5'-GTG TGG TGA TAA AGC CGT GC-3' |
| *Rpl32* | Forward 5'-CGC AAG TTC CTG GTC CAC A-3' |
|  | Reverse 5'-TGC TGC TCT TTC TAC AAT GGC T-3' |
